# Résultats thérapeutiques des carcinomes nasopharyngés: étude monocentrique à l´hôpital universitaire Fattouma Bourguiba de Monastir en Tunisie

**DOI:** 10.11604/pamj.2021.38.143.15510

**Published:** 2021-02-09

**Authors:** Amel El Korbi, Sarra Ben Tkhayat, Rachida Bouatay, Mehdi Ferjaoui, Naourez Kolsi, Khaled Harrathi, Jamel Koubaa

**Affiliations:** 1 Service d´Otorhinolaryngologie et de Chirurgie Cervico-Faciale, Centre Hospitalier Universitaire Fattouma Bourguiba, Monastir, Tunisie,; 2 Unité de Recherche, Qualité et Sécurité des Soins (UR12SP41), Université de Monastir, Monastir, Tunisie

**Keywords:** Carcinome, nasopharynx, survie, pronostic, Carcinoma, nasopharynx, survival, prognosis

## Abstract

**Introduction:**

les carcinomes nasopharyngés (CNP) sont relativement fréquents dans le pourtour méditerranéen. La survie a été largement améliorée avec les nouvelles techniques d´irradiation et les nouvelles molécules de chimiothérapie. L´objectif de notre étude est d´analyser les résultats thérapeutiques et décrire les facteurs pronostiques des CNP.

**Méthodes:**

il s´agit d´une étude rétrospective menée entre janvier 1995 et décembre 2014, des observations de patients traités et suivis dans notre service pour un carcinome du nasopharynx. Le recueil des données a été réalisé à partir d´une fiche type. L´analyse statistique a été réalisée à l´aide du logiciel SPSS. La survie a été calculée selon la méthode de Kaplan-Meier.

**Résultats:**

soixante-treize cas de CNP ont été colligés. L´âge moyen était de 48 ans. Une prédominance masculine a été notée. La majorité des tumeurs (56%) étaient localement avancées (T3-T4). Après un recul moyen de 45,5 mois, le taux de récidives locorégionales était de 19,4%. Trois patients (4%) ont présenté des métastases osseuses. La survie globale à 5 ans était de 65%. Les facteurs influençant positivement la survie globale à l´analyse étaient le stade TNM précoce et le fractionnement de 1,8 Grays/séance de la radiothérapie. Les taux de récidives locales et ganglionnaires étaient respectivement de 13,8% et 5,5%, essentiellement pour des tumeurs classées T4 N2. Les séquelles thérapeutiques étaient dominées par les otites séromuqueuses (42,7%), le trismus (38,7%) et la xérostomie (32%).

**Conclusion:**

la survie globale des CNP s´est améliorée particulièrement avec l´avènement de la radio-chimiothérapie concomitante. Néanmoins, la toxicité tardive invalidante demeure non négligeable. Les facteurs de bon pronostic retrouvés dans cette étude étaient le stade tumoral précoce ainsi que la modalité de fractionnement de la radiothérapie.

## Abstract (en)

**Introduction:**

nasopharyngeal carcinomas (NPCs) are relatively common in the Mediterranean basin. Survival has been greatly improved by new radiation techniques and new molecular-targeted chemotherapy. The purpose of our study was to analyze the therapeutic outcomes and to describe the prognostic factors of NPCs.

**Methods:**

we conducted a retrospective study of patients with nasopharyngeal carcinoma on treatment and follow-up in our department between January 1995 and December 2014. Data were collected using a standardized sheet. Statistical analysis was performed using SPSS software. Kaplan-Meier method was used to determine patient´s overall survival.

**Results:**

seventy-three cases of NPC were reported. The average age of patients was 48 years. A male predominance was noted. The majority of cancers (56%) were locally advanced tumors (T3-T4). After an average follow-up of 45.5 months, the rate of locoregional recurrences was 19.4%. Three patients (4%) had bone metastases. Five-year overall survival was 65%. Factors positively influencing overall survival were early-stage tumor (TNM classification) and 1.8 Gy single fraction radiotherapy. Local and lymph node recurrence rates were 13.8% and 5.5% respectively, mainly for T4 N2 tumors. Therapeutic sequelae were dominated by seromucous otitis (42.7%), trismus (38.7%), and xerostomia (32%).

**Conclusion:**

overall survival in patients with NPC has improved, in particular with the advent of concomitant radiotherapy and chemotherapy; however, disabling late toxicity is significant. Good prognostic factors reported in this study include early tumor stage as well as radiotherapy fractionation.

## Introduction

Les carcinomes nasopharyngés (CNP) sont relativement fréquents dans le pourtour méditerranéen qui représente une zone d´endémicité intermédiaire pour ces cancers [1]. Ils constituent un problème de santé publique vu leur impact thérapeutique et évolutif. Ils présentent des difficultés diagnostiques, du fait d´une symptomatologie clinique d´emprunt. En effet les adénopathies cervicales et les signes otologiques sont inaugurateurs dans plus de 50% des cas [1]. Le carcinome indifférencié type nasopharyngé (UCNT), forme histologique la plus fréquente, se distingue par des particularités épidémiologiques et biologiques du fait de sa répartition bimodale selon l´âge avec une prédominance masculine et son étroite relation avec le virus Epstein Barr (EBV) [1]. La radiothérapie constitue la base du traitement. Son association à la chimiothérapie a montré des bénéfices dans les formes localement avancées [1]. Les principaux facteurs pronostiques des CNP largement décrits dans la littérature incluaient la classification TNM, le volume tumoral, le taux plasmatique de l´ADN viral de l´EBV et le schéma thérapeutique. Nous nous proposons à travers ce travail d´analyser les résultats thérapeutiques de décrire les différents facteurs influençant la survie globale des patients atteints de CNP.

## Méthodes

Nous avons réalisé une étude rétrospective sur une période de 20 ans (janvier 1995-décembre 2014) dans le service d´Otorhinolaryngologie (ORL) et de Chirurgie Cervico-Faciale (CCF) du CHU Fattouma Bourguiba de Monastir en Tunisie. Ont été inclus dans notre étude, toutes les observations des patients suivis et traités pour carcinome du nasopharynx authentifié histologiquement. Ont été exclues de notre étude tous les cas où les dossiers médicaux étaient non exploitables. Le recueil des données a été réalisé à partir d´une fiche de renseignement type. Tous les patients ont eu une biopsie tumorale avec étude anatomopathologique. Selon la classification histologique de l´organisation mondiale de la santé, 93,2% des tumeurs étaient des UCNT (OMS type III) et 6,8% étaient des carcinomes épidermoïdes bien différencié (OMS type I). Le bilan d´extension locorégionale compotait un examen ORL complet, une tomodensitométrie (TDM) du cavum et/ou une imagerie par résonance magnétique (IRM) du cavum. Le bilan d´extension à distance comportait une radiographie du thorax, une échographie abdominale et une scintigraphie osseuse. La classification TNM de l´Union Internationale Contre le Cancer (UICC) de 2010 a été utilisée. En effet, les stades des tumeurs diagnostiquées avant l´année 2010 ont été réévalués selon la classification TNM de 2010.

Les protocoles thérapeutiques établis dans le cadre de réunions de concertations pluridisciplinaires, variaient en fonction du stade tumoral (chimiothérapie néoadjuvante suivie de radiothérapie, chimio-radiothérapie concomitante, radiothérapie exclusive ou chimiothérapie seule). La dose de radiothérapie délivrée variait entre 65 et 74 Gray (Gy) sur le lit tumoral et entre 45 et 74 Gy sur les aires ganglionnaires cervicales. A la fin du traitement, les patients ont été suivis à notre consultation externe tous les 3 mois pendant 2 ans, puis tous les 6 mois pendant 3 ans. Au-delà de 5 ans, la surveillance était annuelle. La réponse thérapeutique a été appréciée par l´examen endoscopique du cavum (avec biopsie à la moindre lésion suspecte), l´examen des aires ganglionnaires et les examens radiologiques (TDM et/ou IRM du cavum). Un bilan biologique thyroïdien a été demandé tous les ans. La toxicité thérapeutique a été évaluée régulièrement lors de ces consultations. Les courbes de survie ont été calculées selon la méthode de Kaplan-Meier. Le test Log-Rank a été utilisé pour l´étude comparative univariée des facteurs pronostiques. Toutes les analyses statistiques ont été réalisées grâce au logiciel SPSS. Le seuil de significativité retenu pour l´ensemble des statistiques était de 0,05.

## Résultats

Soixante-treize cas de CNP ont été colligés à partir de notre étude. L´âge moyen de nos patients était de 48 ans avec des extrêmes allant de 6 à 77 ans. Douze pour cent des patients étaient âgés moins de 20 ans. Une prédominance masculine a été observée avec un sexe ratio de 2,47 (Tableau 1). Au moment du diagnostic, 56% des tumeurs étaient localement avancées (T3-T4). L´atteinte ganglionnaire a été retrouvée dans 75% (N1: 19%, N2: 53%, N3: 3%). La tumeur était d´emblée métastatique chez huit patients, dont trois patients présentaient une double localisation. Le siège métastatique de prédilection était l´os dans 63,6% des cas, suivi du foie (27,2%) et des poumons (9,1%) (Tableau 1).

**Tableau 1:** caractéristiques des patients

Caractéristiques des patients	Nombre de patients n (%)
**Sexe**	
Masculin	**52**(71,2)
Féminin	**21**(28,8)
**Age**	
≤20 ans	**9** (12,3)
>20 ans	**64** (87,7)
**Histologie**	
OMS type III	**68**(93,2)
OMS type I	**5** (6,8)
Classification T	
T1	**21**(29)
T2	**11**(15)
T3	**10**(14)
T4	**31**(42)
Classification N	
N0	**18**(25)
N1	**14**(19)
N2	**39**(53)
N3a	**2** (3)
Métastase	
M0	**65**(89)
M1	**8**(11)
Localisation	
Osseuse	**7**(63,6)
Hépatique	**3**(27,3)
Pulmonaire	**1** (9,1)
Stade TNM	
I	**2**(2,7)
II	**9**(12,3)
III	**27**(37)
IV A	**26**(36)
IV B	**1**(1)
IV C	**8**(11)

Seuls soixante-douze patients ont été traités. En effet un cas de décès est survenu avant le début du traitement. Les protocoles thérapeutiques étaient une radiothérapie exclusive chez 3 patients, une chimiothérapie première suivie de radiothérapie chez 33 patients, une radio-chimiothérapie concomitante chez 35 patients et une chimiothérapie seule chez un seul patient ayant une double métastase osseuse et hépatique (Tableau 2). Un fractionnement classique à raison de 2 Gy/séance, 5 séances/semaine a été adopté chez 62 patients et de 1,8 Gy/séance, 5 séances/semaine chez 9 patients. L´étalement moyen de la radiothérapie était de 64,3 jours (36-106 jours). Le délai moyen entre la chimiothérapie néoadjuvante et la radiothérapie était de 49,5 jours, avec des extrêmes de 13 à 190 jours.

**Tableau 2:** protocoles thérapeutiques

Protocole	Nombre de patients	Stades	Nombre de patients
RT exclusive	3	**I**	2
		**II**	1
	33	**II**	2
		**III**	13
CT néoadjuvante + RT		**IV A**	11
		**IV B**	1
		**IV C**	6
		**II**	6
CT- RT concomitante	35	**III**	14
		**IV A**	15
CT	1	**IV C**	1

**Figure 1:**
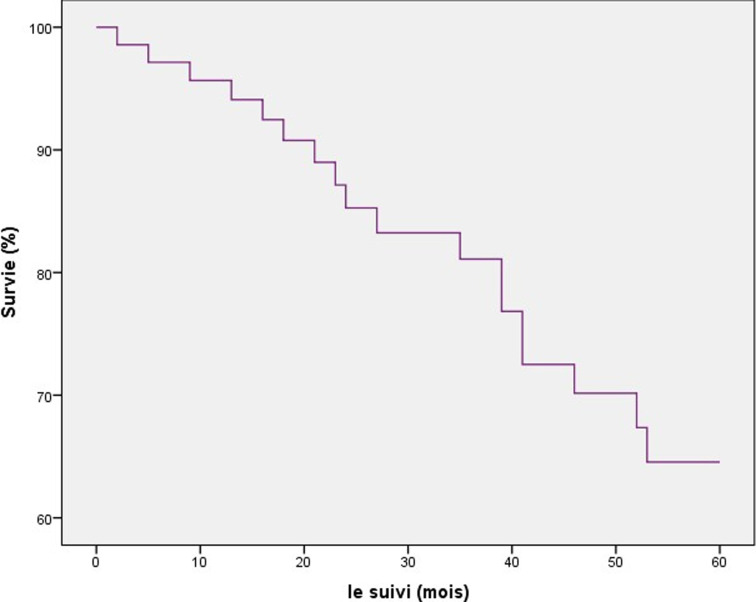
courbe de la survie globale

Une rémission complète a été obtenue chez 60 patients (83,3%), elle était partielle chez 13,8% patients. Une stabilisation de la maladie a été constatée chez deux patients (2,7%). Après un recul moyen de 45,5 mois, un patient a été perdu de vue, quatorze patients ont présenté des récidives locorégionales (19,4%) et 3 patients (4%) ont vu se développer des métastases osseuses. Soixante-dix pour cent des récidives locales, ont apparu au cours des deux premières années. Le délai moyen d´apparition des métastases était de 29 mois. L´os était le siège de prédilection des rechutes à distance. Le taux de contrôle local à 3 ans était de 29%. La survie globale (SG) à 5 ans était de 65% (Figure 1). La toxicité aigüe était dominée par la radiodermite (73,3%) et la radiomucite (69,3%). Les séquelles thérapeutiques étaient dominées par l´otite séromuqueuse (42,7%), le trismus (38,7%) et la xérostomie (32%). L´analyse statistique a montré que le stade TNM précoce ainsi que le fractionnement de la radiothérapie à la dose de 1,8 Gy/séance constituaient des facteurs de bon pronostic en termes de taux de survie globale (Tableau 3).

**Tableau 3:** facteurs pronostiques des carcinomes nasopharyngés

	Nombre de patients	Survie globale à 5 ans (%)	p
**Âge**			
≤ 20 ans	9	65	0,6
> 20 ans	64	62	
**Sexe**			
Masculin	52	57,5	0,28
Féminin	21	77	
**Délai de consultation**			
=< 6mois	46	79	0,58
>6 mois	27	60	
**Atteinte des nerfs crâniens**			
Non	40	71	0,66
Oui	33	13	
**Aires ganglionnaires**			
Pas d´atteinte	40	100	
IIa	9	100	0,24
IIb	10	67	
III	3	72	
V	20	40	
**Latéralité ganglionnaire**			
Unilatérale	20	73	0,53
Bilatérale	15	54	
**Atteinte parapharyngée**			
Oui	8	62,5	0,85
Non	65	65	
**Classification T**			
T1	21	78	0,48
T2	11	80	
T3	10	66	
T4	31	44	
**Classification N**			
N0	18	100	0,75
N1	14	68	
N2	39	77	
N3a	2	56	
**Stade TNM**			
I	2	100	
II	9	100	0,04
III	27	75	
IV A	26	57	
IV B	1	71	
IV C	8	13	
**Méthodes thérapeutiques**			
RT	3	66,6	0,99
RT-CT concomitante	35	52	
CT néoadjuvante + RT	33	43	
**Dose reçue au niveau du cavum**			
65 Gy	1	100	0,07
70,4 Gy	11	80	
74 Gy	59	62	
**Fractionnement de la RT**			
1,8 Gy/ séance	9	87,8	0,04
2 Gy/ séance	62	62	
**Etalement de la RT**			
≤ 60 jours	37	73	0,38
> 60 jours	34	59	
**Délai entre CT néoadjuvante et RT**			
=< 1 mois	11	60	0,32
>1mois	22	52	

## Discussion

Les CNP se caractérisent par la fréquence des formes localement évoluées. Dans notre série, 56% des tumeurs étaient classées T3/T4. Ce taux varie entre 32% et 79,5% selon les différentes séries de la littérature [2-7]. Une atteinte ganglionnaire importante (N2 ou N3) y était souvent associée, témoignant du caractère lymphophile de ces cancers. Son taux varie entre 33,3% et 69% selon les données de la littérature [2-7]. Dans notre série, 56% des atteintes ganglionnaires étaient classées N2/N3. Ces tumeurs sont aussi dotées d´un haut potentiel métastatique, que ce soit au moment du diagnostic ou lors de lors de l´évolution de la maladie. Dans la littérature, les métastases diagnostiquées au moment du diagnostic variaient entre 6,6% et 24% [3, 8-10]. Dans notre série, ce taux était de 11% (8 patients) avec associations de deux localisations métastatiques chez trois patients (osseuse et hépatique dans deux cas et osseuse et pulmonaire dans un cas).

L´objectif du système de classification TNM est de planifier une stratégie thérapeutique, établir un pronostic et évaluer les résultats thérapeutiques afin d´assurer une meilleure prise en charge pour les patients. Depuis plusieurs années, on assiste à une amélioration progressive de la survie des CNP, notamment en Tunisie, suite à une meilleure prise en charge multidisciplinaire et les protocoles thérapeutiques notamment la radio-chimiothérapie concomitante [2]. En effet, la survie globale à 5 ans, rapportées par plusieurs études randomisées varie entre 49,7% et 78,4% [3,4,10,11]. Dans notre série, la SG à 5 ans était de 65%. Les facteurs pronostiques étaient essentiellement le stade TNM et le fractionnement de la radiothérapie. En effet, un stade TNM précoce (I, II) et un fractionnement à 1,8 Gy/séance et 5 séances/semaine étaient corrélés à de meilleurs taux de survie avec des différences statistiquement significatives. Selon la littérature, des facteurs pronostiques épidémio-cliniques significatifs pour la survie globale à 5 ans ont été déterminés. En effet, dans la plupart des études, l´âge jeune (≤20 ans) ressort comme un facteur de bon pronostic malgré la fréquence des formes évoluées [1,2]. De même, le sexe féminin a été corrélé à de meilleurs taux de survie globale à 5 ans [3,11,12]. Sur le plan clinique, l´atteinte des paires crâniennes, traduisant souvent des tumeurs évoluées, a été largement considérée comme un facteur de mauvais pronostic [13,14]. Le volume tumoral (T) ainsi que l´envahissement ganglionnaire (N) constituent des facteurs pronostiques potentiels pour la survie globale [8]. La majorité des auteurs rapportaient un taux de survie globale à 5 ans d´autant plus élevé que la tumeur est localement peu évoluée et que l´envahissement ganglionnaire [8,14]. Selon la série de Chen *et al*. [11], la survie globale à 5 ans pour les tumeurs classées T3 et T4 était respectivement de 69,12% et 58,96% avec une différence significative (p=0,035) et de 74% contre 29,4% pour les stades N2 et N3 (p=0,0009). Le stade TNM a été considéré comme facteur pronostique majeur, qui tient compte du volume tumoral, de l´atteinte ganglionnaire et des métastases [2,8,15]. Plus le stade TNM était avancé, plus mauvais était le taux de SG. Nos résultats concordaient avec ceux de la littérature. En effet, dans notre série, la SG à 5 ans était respectivement de 100% et 13% pour les stades I/II et IV C avec une différence significative (p=0,04).

Le traitement des CNP repose essentiellement sur la radiothérapie, du fait de l´impossibilité d´obtenir une résection carcinologique satisfaisante étant donnée sa localisation anatomique profonde et proche de la base du crâne [2,3,16,17]. Ces cancers sont également chimiosensibles, notamment l´UCNT, et ceci constitue la base des protocoles thérapeutiques visant à associer ces deux modalités thérapeutiques [7,18]. La radio-chimiothérapie concomitante a prouvé sa supériorité à la radiothérapie exclusive et à la chimiothérapie adjuvante, en améliorant le taux de survie globale à 5 ans de 4 à 6%. Elle peut être ainsi considérée comme le « Gold Standard » du traitement des tumeurs évoluées [10,19-22]. La dose d´irradiation reçue par le cavum ainsi que l´étalement de la radiothérapie, sont considérés des facteurs pronostiques influençant plutôt le taux de contrôle local que celui de la survie globale [23]. La modification du fractionnement de la radiothérapie a prouvé son intérêt dans les tumeurs de la sphère ORL. Cependant, dans les cancers du cavum, son influence en termes de survie globale est controversée [2,8]. Dans notre série la survie globale à 5 ans était significativement meilleure (p=0,04) après une radiothérapie à 1,8 Gy/séance (87,8%) comparativement à un fractionnement de 2 Gy/séance, 5 séances/semaine (62%). Ainsi, les majeurs facteurs pronostiques influençant la survie globale des CNP sont représentés essentiellement par le volume tumoral, l´envahissement ganglionnaire, le stade TNM, la radio-chimiothérapie concomitante et le fractionnement de la radiothérapie. L´amélioration du taux de contrôle locorégional et à distance demeure l´objectif majeur des protocoles thérapeutiques, néanmoins les rechutes métastatiques restent un écueil thérapeutique influençant négativement le pronostic des CNP.

## Conclusion

Les résultats thérapeutiques des carcinomes nasopharyngés sont prometteurs et ne cessent de s´améliorer. Les facteurs pronostiques les plus influent sur le taux de survie globale sont représentés par le volume tumoral, l´envahissement ganglionnaire, le stade TNM, la radio-chimiothérapie concomitante et le fractionnement de la radiothérapie. On espère dans l´avenir, avec les nouvelles molécules de chimiothérapie anti-cancéreuses, les thérapies ciblées (anti-*Epidermal Growth Factor* ou anti-angiogenèse) et l´immunothérapie ainsi que les nouvelles techniques d´irradiation (IMRT: *Intensity Modulated Radiotherapy*) améliorer le taux de survie globale des carcinomes nasopharyngés en Tunisie.

### Etat des connaissances sur le sujet

- La survie globale du cancer du cavum a été nettement améliorée par la radio-chimiothérapie concomitante;
- La dose d´irradiation reçue par le cavum ainsi que l´étalement de la radiothérapie, sont considérés comme facteurs pronostiques influençant le taux de contrôle local;
- Les facteurs pronostiques les plus influents sur la survie globale sont: l´âge, le sexe, l´atteinte des paires crâniennes, le volume tumoral (T) ainsi que l´envahissement ganglionnaire (N).

### Contribution de notre étude à la connaissance

- La survie globale à 5 ans reste satisfaisante (65%) bien que 85% de nos patients étaient diagnostiqués à des stades avancés (III/IV);
- L´étalement de la radiothérapie est un facteur pronostique pour la survie globale.
